# Cardiovascular disease: The rise of the genetic risk score

**DOI:** 10.1371/journal.pmed.1002546

**Published:** 2018-03-30

**Authors:** Joshua W. Knowles, Euan A. Ashley

**Affiliations:** Center for Inherited Cardiovascular Disease, Stanford University, Stanford, California, United States of America

## Abstract

In a Perspective, Joshua Knowles and Euan Ashley discuss the potential for use of genetic risk scores in clinical practice

## Summary points

- Use of risk factors for decision-making in cardiovascular disease has a long history in medicine.
- Early attempts to augment traditional risk factors with genetic risk scores were hampered by too little understanding of the genetic basis of complex cardiovascular disease.
- Newer studies based on hundreds of thousands of people and millions of genetic variants indicate that genetic risk scores can now outperform traditional risk factors in risk prediction.
- We propose the time has come to incorporate genetic risk scores into clinical practice.
- Studies should focus on the most appropriate way to do this to maximize benefit for our patients.

“[*However*,*] epidemiologic information has accumulated which now allows the physician to recognize certain characteristics of increased risk in patients he [sic] sees in his practice*. *Some of these characteristics have been convincingly demonstrated*, *others are still under investigation*. *More precise identification will undoubtedly be possible in the future*.”—William Kannel, Director, Framingham Heart Study [1]

In a classic paper [1], Kannel reported the early results of the longitudinal Framingham Heart Study, demonstrating the identification of factors (for which he coined the term “risk factors”) that “*precede* the development of overt coronary heart disease in humans”. Later, the Framingham Risk Score was formalized to include age, sex, diabetes, smoking status, total cholesterol, high-density lipoprotein (HDL) cholesterol, and blood pressure [2], providing a framework for cardiovascular disease (CVD) risk assessment to which all others are compared. Intuitively, the burden of these risk factors accumulates over time (e.g., pack years of smoking or years of hypertension), and some newer risk models allow input of risk factor data from multiple time points [3].

While repeated measurement of CVD biomarkers such as total cholesterol may improve risk prediction, lifelong exposure to CVD risk factors is better captured by genetic susceptibility [4]. Thus, the quest to improve risk prediction for CVD has naturally come to focus on the development of genetic risk scores [5]. This has only been possible because of robust, replicable findings from genome-wide association studies (GWAS) in extremely large cohorts [6,7]. Early genetic risk scores, based on relatively few single-nucleotide variants, showed a consistent ability to identify those in the highest strata of risk [8,9], with some improvement in “reclassification” of risk. This interest in risk prediction led to an increased focus on the tools for judging utility with a return to prominence of metrics like the C statistic and the proposal of newer metrics, such as the integrated discrimination improvement (IDI) and the net reclassification index (NRI), specifically aimed at judging the merit of adding new factors (i.e., genetic markers) to existing scores [10]. Although the focus of many hundreds of articles, these newer metrics have been criticized for too highly rating poorly fitted risk models and for showing improvement in models with a new biomarker that adds no new information [11–13]. Around 2009, there was also criticism of the common variant studies for failing to find “missing” heritability [14], and the lack of robust risk prediction from discovered variants fed into an overall narrative that genomics was underperforming relative to its hype [15].

Yet it was clear that this was chiefly a problem of study size. While human clinical trials have historically recruited hundreds or thousands of individuals, the genomics community realized that studies with hundreds of thousands to millions of participants would be required to provide the power necessary to fuel discovery of the larger proportion of heritability. This realization ushered in a new era of data sharing. Today, as a result of large-scale collaboration, meta-analysis, and the emergence of national projects such as the United Kingdom Biobank [16,17], there are GWAS of common variants drawing on more than 1 million individuals [18]. Such studies, as modeling would predict [19], are beginning to demonstrate that genetic factors provide robust and powerful risk estimation across diseases that is additive to traditional risk factors [20–22]. Indeed, as the idea that rare variation (synthetic or otherwise) could explain much of the missing heritability of common disease fell in favor [23] and out again [24], the realization dawned that still-too-small studies and overzealous correction of multiple testing had left significant signal in the noise. This stimulated the idea of using a much broader array of variants in a polygenic score. Khera and colleagues [21] used 6.6 million variants, while Inouye et al. [22] used 1.7 million variants as predictors, and both studies demonstrate the ability to identify a group in the upper echelon of genetic risk with a hazard of greater than 4. In particular, the meta genetic risk score (metaGRS) had a higher C-index for incident coronary artery disease than any single traditional risk factor, including smoking, diabetes, hypertension, and body mass index [22]. In that study, the addition of the genetic score to a combination of conventional risk factors increased the C-index by 3.7%. Drawing on multiple interacting mechanisms, it is not surprising that much of the signal of genetic risk scores overlaps traditional risk factors and mechanisms. But the potential of genetic approaches is emphasized by a component of independence, illustrated by the ability to improve on a C-index derived from conventional risk factors alone.

Thus, despite early criticism, most recent genetic risk scores have demonstrated significant improvements in performance for risk prediction in CVD [25]. Given these advantages, it is reasonable to ask whether such scores have the potential to significantly improve multimorbidity assessment for diseases where risk assessment has been routine, especially as the costs of genome-wide genotyping now fall below US$100 per person. Indeed, because genotyping chips survey common variants across the entire genome, reflecting risk for hundreds of conditions besides CVD, it is possible to simultaneously predict risk of multiple diseases with a single “test.” Cardiometabolic scores [26,27] can be combined [28], or estimates can be made, for dozens of diseases, including—as we reported [29]—from whole-genome sequencing.

A critical aspect of the utility of any predictive score is its impact on clinical management. Since Kannel’s coining of the term, risk prediction has been leveraged for management decisions in medicine. Recent guidelines on hypertension [30] and hypercholesterolemia [31,32] emphasize the role of risk estimation in therapeutic decision-making, particularly for patients with intermediate risk. Yet, whereas cholesterol levels can be lowered through therapy and individuals can stop smoking, what is the specific “answer” to a high genetic risk score? Khera and colleagues [20] provided one answer in a study demonstrating that lifestyle factors are capable of abrogating genetic risk, elegantly underlining the universality of the benefits of diet and exercise while providing a defense for the concern that patients who discover they are at high genetic risk will view that deterministically and be less inclined to lifestyle change (something that has always remained hypothetical [33]). Another recent study has shown that genetic risk for high blood pressure can be mitigated by a healthy lifestyle [34]. Additional data are needed to address the converse concern that individuals shown to have a “protective” genetic background will feel less inclined to maintain a healthy lifestyle. In this regard, the best outcomes are in those individuals that have both a favorable genetic susceptibility and healthy lifestyle [20].

So if polygenic risk scores now outperform traditional risk factors in univariate prediction, augment the C statistics of traditional risk factors taken as a whole, can be implemented for minimal cost, and are targets for intervention, is it not time to incorporate them into clinical practice?

Despite the increasingly well-demonstrated value of the genetic risk scores, few studies have focused on the practical aspects of incorporating scores into clinical practice. Although the benefit of delivering traditional risk factors to physicians and patients has never itself been tested in a randomized controlled trial, the traditional risk score, based on data already gathered, is effectively free to the healthcare system. While there remains an additional cost for genetic scores, albeit modest, it is reasonable to require an outcome benefit to be demonstrated before arguing for adding to medical expenditure. In a small pilot randomized controlled study, we showed the feasibility of delivery of a genetic risk score in a clinical environment [35–37]. While we did not demonstrate that the score led to an improvement in patient adherence to guideline-based therapeutic advice, others have shown that the incorporation of a genetic risk score into clinical care may increase statin usage (mostly through increased statin prescriptions) [38]. We would note that similar challenges in changing behavior despite improving risk prediction have been reported in studies of coronary calcium, carotid ultrasound, and coronary computed tomography (CT) scans [39–41]. However, as we become more sophisticated in delivery of information to “activate” positive behavioral changes, these results are expected to improve. Digital approaches may offer one avenue for improvement: for example, there are now smartphone studies of cardiovascular risk that incorporate genotype data, as well as studies focused specifically on returning genetic risk scores to participants [42–44].

In an additional wrinkle, if the genetic risk score could be calculated from preexisting data, the cost to the healthcare system would be zero, and few would argue that we should not look to refine traditional scores with genetic data. The highly computable nature of genotype data makes for straightforward implementation and future refinement of genetic risk scores when more data become available [45]. Indeed, the ability to create scores across multiple diseases was attractive for direct-to-consumer genetic testing companies who started offering such estimates for multiple diseases and traits many years ago. Early versions received technical criticism based on the small numbers of variants used and the variation between providers in the creation and interpretation of scores. However, this technical criticism was secondary to more general uncertainty over the direct-to-consumer model [46]. Today, with increasing interest from the public and increasing acceptance—at least in the United States from the Food and Drug Administration (FDA)—of consumer-focused tests, the environment is primed for delivery and testing of multimodal risk scores for millions of individuals through direct-to-consumer services utilizing laboratories accredited under the Centers for Medicare and Medicaid Services Clinical Laboratory Improvement Amendments (CLIA) standard. Healthcare systems and academic clinicians should work together with these companies to ensure standards and transparency in the safe and effective translation of these data for the public good [47].

We believe there are strong reasons to now consider incorporation of genetic risk scores into clinical practice. But questions remain. Since genetic information is viewed as more sensitive than that of other risk factors and since genetic risk does not result from an individual choice, some countries have chosen to separately protect genetic information from discrimination by health insurers or employers. The US Genetic Information Non-Discrimination Act of 2008 [48] includes both of those protections but excludes protection from life insurance discrimination. As such, before testing for a genetic risk score, individuals should receive education beyond that which a treating physician or nurse might be comfortable delivering. The scale of common disease means that the genetic counselor workforce could not meet the demand of delivering counseling for common disease risk scores. Brief video education has, however, been shown to be engaging and compelling, even for much more complex concepts in genetics [49]. Decision support would also be required for physicians and nurses incorporating scores into clinical management. Another challenge that has existed since the earliest use of risk factors in clinical medicine is that of unmeasured factors. A good prognostic score produces a prediction that, on a population level, has acceptable test characteristics. It cannot, however, speak to unmeasured factors in the individual. In the genetic era, this is most relevant for rare variation. It is possible, for example, for an individual to have a common variant risk score that places them in the lowest quintile for risk, but for that individual also to harbor a rare variant of large magnitude in, for example, the gene *LDLR* that overwhelms the common risk and places them instead in the highest quintile—or even gives them a Mendelian disease. The most proximal answer to this issue is education: unmeasured factors are not a challenge specific to genetic risk scores. The more distant answer—though one with its own nuanced challenge—is to use genome sequencing, a future imagined almost a decade ago [28,29,50].

In conclusion, through collaboration and data sharing, genetic studies of common diseases now allow genetic risk scores that predict future diseases better than traditional risk factors. As millions around the world already have this data in hand, and as the cost of generating this data falls further towards the cost of a daily cup of coffee for just one week, we propose that the time has finally come to build for the testing and incorporation of genetic risk scores into clinical practice.

## Abbreviations

CLIA: Clinical Laboratory Improvement Amendments
CT: computed tomography
CVD: cardiovascular disease
FDA: Food and Drug Administration
GWAS: genome-wide association studies
HDL: high-density lipoprotein
IDI: integrated discrimination improvement
metaGRS: meta genetic risk score
NRI: net reclassification index
